# First clinical implementation of audiovisual biofeedback in liver cancer stereotactic body radiation therapy

**DOI:** 10.1111/1754-9485.12343

**Published:** 2015-08-06

**Authors:** Sean Pollock, Regina Tse, Darren Martin, Lisa McLean, Gwi Cho, Robin Hill, Sheila Pickard, Paul Aston, Chen‐Yu Huang, Kuldeep Makhija, Ricky O'Brien, Paul Keall

**Affiliations:** ^1^ Radiation Physics Laboratory, Sydney Medical SchoolThe University of SydneySydneyNew South WalesAustralia; ^2^ Department of Radiation OncologyChris O'Brien LifehouseSydneyNew South WalesAustralia

**Keywords:** abdomen, intervention, physics, radiation oncology imaging, radiation oncology, respiratory

## Abstract

This case report details a clinical trial's first recruited liver cancer patient who underwent a course of stereotactic body radiation therapy treatment utilising audiovisual biofeedback breathing guidance. Breathing motion results for both abdominal wall motion and tumour motion are included. Patient 1 demonstrated improved breathing motion regularity with audiovisual biofeedback. A training effect was also observed.

## Introduction

**Figure 1:**
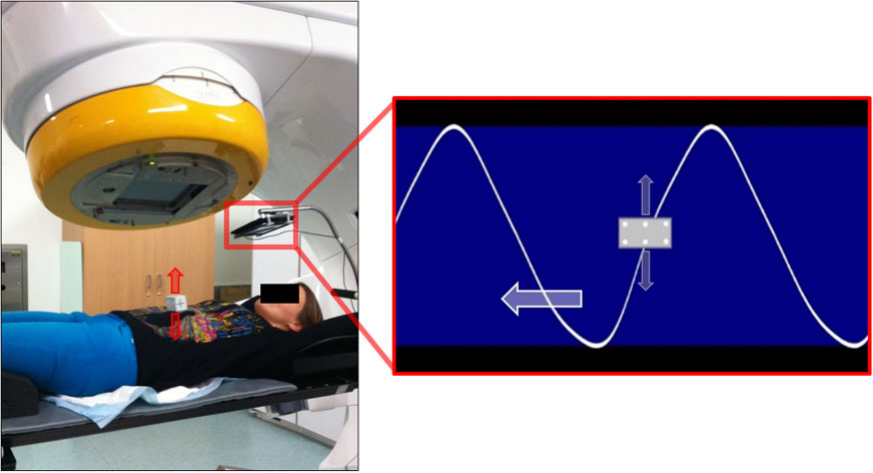
Study setup in the linac bunker with the Real‐time Position Management (RPM) marker block and patient display (left). AVB (audiovisual biofeedback) interface (right).

Liver tumours are highly mobile due to their proximity to the thoracic diaphragm. When a patient's breathing motion is irregular, it exacerbates both systematic and random errors which compromise the accuracy of radiation therapy.^1^, ^2^ To reduce these errors, breathing guidance strategies have been investigated to facilitate stable and regular breathing.^3^, ^4^ This study represents a milestone in breathing guidance investigations as it addresses a gap in the literature by assessing the impact of the breathing guidance system, audiovisual biofeedback (AVB), on intra‐ and inter‐fraction liver tumour motion, via fiducial marker surrogacy, in liver cancer patients undergoing stereotactic body radiation therapy (SBRT). The AVB system, shown in Figure 1, utilises audio and visual prompts to guide the patient to breathe regularly. External breathing motion from the Real‐time Position Management (RPM) system (Varian Medical Systems, Palo Alto, CA, USA) of the patient's abdominal wall is shown on the patient display. The marker block moves up as they inhale and down as they exhale. The patient adjusts their breathing such that the marker block stays within the blue region and traces the motion of the waveguide (white wave in Fig. 1).

## Case report

Patient 1 was a 65‐year‐old male with metastatic (recurrent) cholangiocarcinoma and received 36 Gy across 6 fractions using volumetric‐modulated arc therapy‐based SBRT to a 30 mm solitary lesion in segment 8 of the liver. Due to previous liver resection, this patient had pre‐existing surgical clips implanted into his liver, which were utilised for image guidance. He had a number of other comorbidities including bronchiectasis with impaired pulmonary function and was of Karnofsky performance status 1. Prior to treatment planning, a screening procedure was performed to ensure that the most regular breathing condition (free breathing (FB) or AVB) was utilised throughout the patient's subsequent course of SBRT. Breathing motion was monitored for 4 minutes for each of the breathing conditions FB and AVB; at the 2‐minute mark, cone beam CT (CBCT) images were acquired. Determining which breathing condition would be selected was based on the regularity of the 4 minutes of external breathing motion (quantified by the root mean square error (RMSE) in displacement and period); the lower the RMSE, the more regular the breathing motion. Decisions were made *in situ* using a function within the AVB software. Patient 1's screening procedure yielded the decision to utilise AVB for the remainder of their course of SBRT.

**Figure 2:**
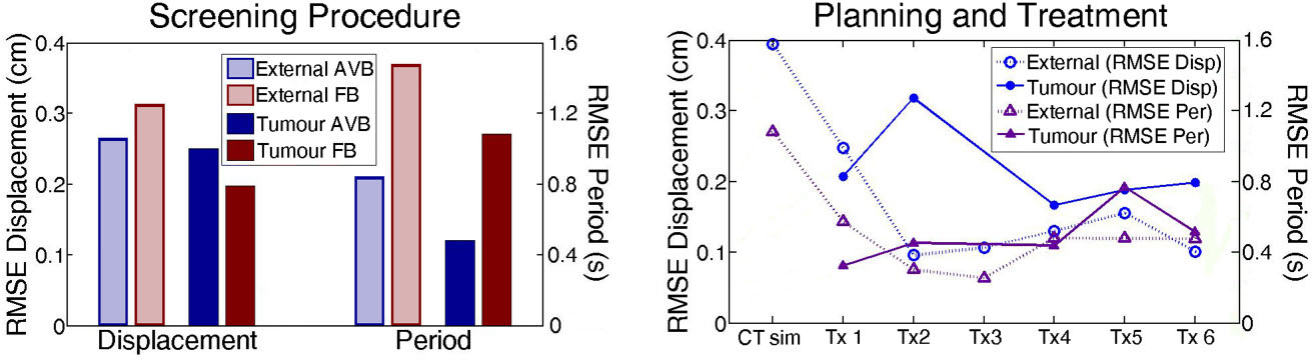
AVB (audiovisual biofeedback) and FB (free breathing) RMSE (root mean square error) results for Screening Procedure (left); and results for AVB across patient 1's course of treatment (right), for RMSE of displacement (RMSE Disp, blue circle markers) and RMSE of period (RMSE Per, purple triangle markers). External motion shown as hollow markers/bars and dotted lines, tumour motion shown as solid markers/bars and unbroken lines.

**Figure 3:**
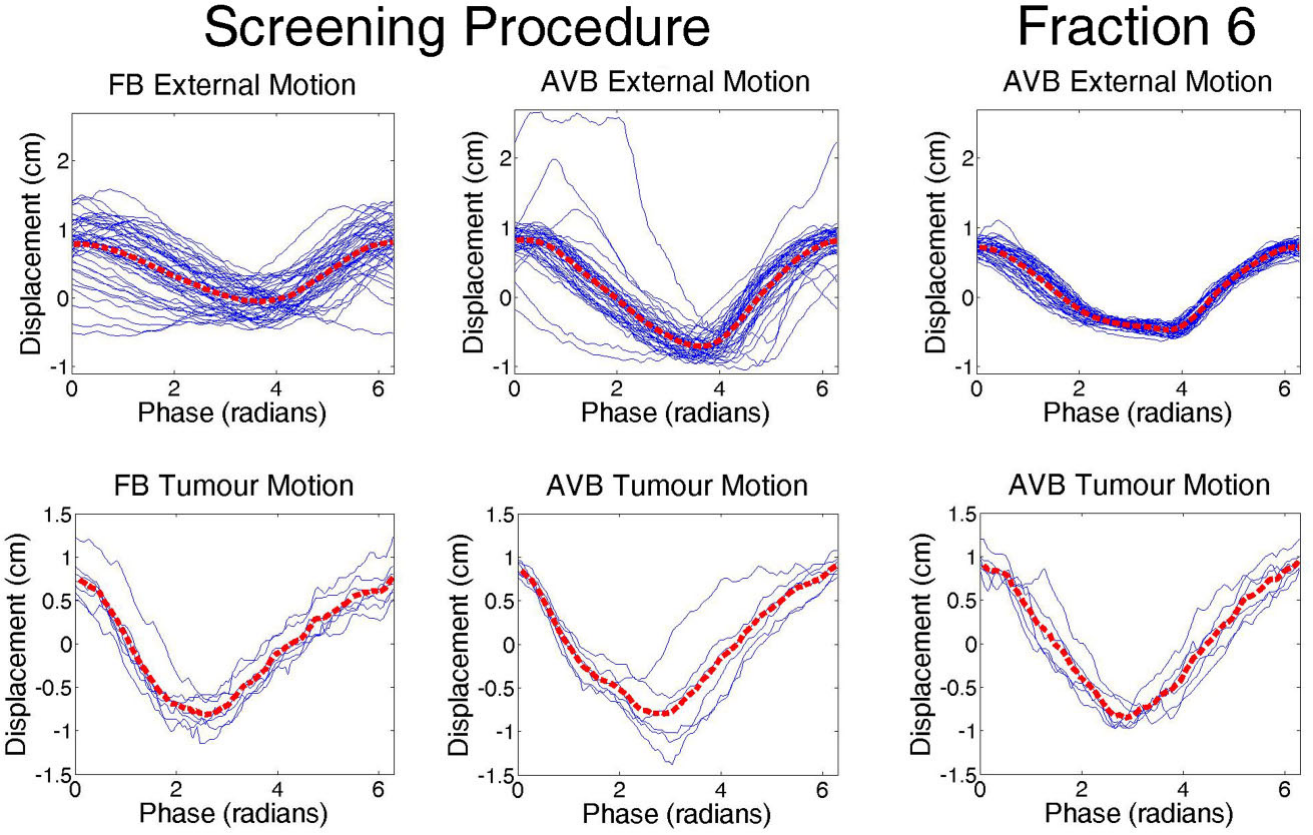
The external motion (top) and tumour (bottom) individual breathing cycles for FB and AVB Decision Sessions (left) and Fraction 6 (right). Unbroken blue lines represent each individual breathing cycle, and the dotted red line is the average cycle.

Patient 1's treatment planning and treatment delivery proceeded as per the currently implemented clinical liver SBRT protocol with the addition of the AVB setup (see Fig. 1). CBCT images were acquired prior to treatment delivery on each day of treatment, motion of the surgical clips was extracted from the CBCT projection images utilising a method developed by Fledelius *et al*.,^5^ as a surrogate for tumour motion. Figure 2 and Figure 3 demonstrate the breathing motion results across patient 1's course of radiotherapy. It was also observed that AVB increased the average range of tumour motion from 1.5 cm for FB, to 1.8 cm for AVB.

## Discussion

This study reported on the first patient recruited into a clinical trial investigating the use of breathing guidance during a course of liver SBRT planning and treatment utilising an initial screening procedure. A training effect was observed, with the patient's breathing motion becoming more regular inter‐fractionally, plateauing at peak regularity around Fraction 3. It was also observed that AVB increased breathing amplitude compared with FB. Given that the AVB waveguide peak‐to‐peak amplitude was set at 1.5 cm and the observed external peak‐to‐peak amplitude was 1.7 cm indicates that Patient 1 ‘over‐shot’ the AVB breathing limits. For future patients in this study further attention will be given to managing breathing motion amplitude and patient training.

In conclusion, the first patient recruited into this study yielded the decision to utilise AVB through their course of SBRT. Patient 1 demonstrated good acceptance of the breathing guide in addition to increasingly regular breathing throughout their course of SBRT.
